# Modulating the *bicoid* gradient in space and time

**DOI:** 10.1186/s41065-021-00192-y

**Published:** 2021-08-17

**Authors:** Xiaoli Cai, Inge Rondeel, Stefan Baumgartner

**Affiliations:** 1 Departmentof Experimental Medical Sciences, Lund University, BMC D10, 22184 Lund, Sweden; 2 Present address: Hubrecht Institute, 3584 CT Utrecht, The Netherlands; 3 Department of Biology, University of Konstanz, 78457 Konstanz, Germany

## Abstract

**Background:**

The formation of the Bicoid (Bcd) gradient in the early *Drosophila* is one of the most fascinating observations in biology and serves as a paradigm for gradient formation, yet its mechanism is still not fully understood. Two distinct models were proposed in the past, the SDD and the ARTS model.

**Results:**

We define novel *cis*- and *trans*-acting factors that are indispensable for gradient formation. The first one is the poly A tail length of the *bcd* mRNA where we demonstrate that it changes not only in time, but also in space. We show that posterior *bcd* mRNAs possess a longer poly tail than anterior ones and this elongation is likely mediated by *wispy* (*wisp*), a poly A polymerase. Consequently, modulating the activity of Wisp results in changes of the Bcd gradient, in controlling downstream targets such as the gap and pair-rule genes, and also in influencing the cuticular pattern. Attempts to modulate the Bcd gradient by subjecting the egg to an extra nuclear cycle, i.e. a 15^th^ nuclear cycle by means of the *maternal haploid* (*mh*) mutation showed no effect, neither on the appearance of the gradient nor on the control of downstream target. This suggests that the segmental anlagen are determined during the first 14 nuclear cycles. Finally, we identify the *Cyclin B* (*CycB*) gene as a *trans*-acting factor that modulates the movement of Bcd such that Bcd movement is allowed to move through the interior of the egg.

**Conclusions:**

Our analysis demonstrates that Bcd gradient formation is far more complex than previously thought requiring a revision of the models of how the gradient is formed.

## Introduction

The formation of the Bicoid (Bcd) gradient in *Drosophila* is one of the most fascinating observations in biology that has intrigued scientists for many decades [1]. Originally discovered as a *Drosophila* mutant that affects anterior patterning of the early egg [2], the remarkable Bcd protein gradient became the hallmark that served as paradigm for gradient formation in science for almost three decades [3]. To explain the occurrence of the gradient, a model was first proposed in 1988, the SDD model [1, 3–5]. The 3 letters SDD stand for synthesis, diffusion, degradation. The SDD model proposed that the *bcd* mRNA, held at the tip upon fertilization and later stages of the embryo, would serve as the source for the translation of the Bcd protein. Bcd protein would then diffuse uniformly through the entire embryo to form the gradient, followed by uniform degradation. In 2007, the first doubts on the validity of the model appeared, because the diffusion properties of Bcd were found to be too low to account for establishing a steady-state gradient in the short period of 2 h during egg development [6]. The ARTS model [1, 7] was proposed in 2009 to solve the apparent difficulty of the SDD model in explaining the fast establishment of the gradient. ARTS stands for active RNA transport synthesis. The model was based on earlier observations [8] that the *bcd* mRNA itself formed a gradient. To account for the rapid gradient establishment, a model was proposed that involved active transport of the *bcd* mRNA along microtubules (MTs) at the cortex of the embryo [7]. Since the transport of *bcd* mRNA was shown using in vivo imaging [9, 10] to occur in the oocyte at a speed range of 0.36–2.15 μm/sec, it was conceivable to assume that the egg used the same machinery to transport the mRNA away from the anterior tip to the posterior of the egg. The ARTS model necessitated the existence of a cortical MT network to serve for the transport [7]. An important facet of the ARTS model was that the observed speed of the active transport was fast enough to explain the establishment of the gradient in the given time. It took some time until a report on the proposed microtubular network at the cortex was published [11]. The transient network built up only for a short time during metaphase and early anaphase of each nuclear cycle and after that was immediately degraded again. Henc, it was visible for approximately 2 min during an early nuclear cycle lasting about 10 min [12]. Fahmy K, et al. [11] also identified a MT-minus-end motor protein, Ncd as a player of this transport machinery and essential for the posteriorwards transport of the *bcd* mRNA. In a following report, [13] revealed that the Bcd protein consistently moved along the outermost cortex and never entered the interior of the egg, thus defining the interior yolk as a non-permissive territory for Bcd movement. If drugs compromising the major constituent of the egg’s cytoarchitecture, the microtubules (MTs) or actin were administered, the behavior of the movement of the Bcd protein changed significantly [13]. Upon drug administration, the interior of the egg became permissive, and Bcd moved to the posterior in a broad front, thus conforming to the SDD model [3]. While disrupting microtubules had a significant effect on Bcd movement, compromising actin had an effect on Bcd stability, but was not strongly involved in Bcd movement [13]. Consistent with this definition of the cortex as the major compartment in the early *Drosophila* egg, a recent study on the diffusion of proteins revealed that test proteins always migrated along the cortex, but never entered the yolk [14]. Of note, hints as to the compartmentalization of the early *Drosophila* egg already came from early studies using simple microscopic observations [12]. However, the analysis of the composition and biochemical properties of the inner yolk had been largely neglected in the past, primarily due to technical challenges during the analyses of the optically-dense material which led to sparse information of the interior structure of the early egg [15–18]. Only during recent advances has the use of fluorescent techniques allowed to shed some light into the activities of the inner part of the egg [19].

Based on the fact that early nuclei were never located at the cortex, little information was available in regard to whether or not a microtubular organizing center (MTOC) would exist at the cortex to initiate growth and destruction of the short-lived MT-network. Hints to the existence of a Golgi-based acentriolar microtubule organizing center (aMTOC) came first from vertebrate studies showing that structures at the *trans*-Golgi could serve as a starting point for MT nucleation [20]. Only very recently has [21] shown that Golgi structures do have an impact on *bcd* mRNA localization in the oocyte and the early *Drosophila* embryo, where they depended on components of the *trans*-Golgi compartment. In terms of the localization of Golgi structures in the early *Drosophila* egg, some were shown to be present at the cortex [21–23], but we are far from understanding how the cortical Golgi apparatus of the early *Drosophila* embryo is organized.

Additional theories of nucleating MTs at the cortex were recently proposed in the context of lipid droplets (LD) and yolk granules (YG) and the possibility that LD-associated proteins can exert control on MT nucleation from centrosomes [24]. One of these proteins associated with YGs was shown to be Mauve/LYST. Since YGs are strongly enriched in the cortical layer of the early *Drosophila* embryo, it was conceivable that MTs can also be nucleated from yolk granules and that these structures together with Mauve/LYST constitute another aMTOC at the cortex.

In the past, a number of factors affecting the activity of *bcd*, either at the mRNA or protein level were detected. Each have a distinct mode of interaction with *bcd*. At the mRNA level, the most prominent ones were the RNA-binding proteins Staufen [25], the poly A polymerase Wispy [26], the pseudonuclease Exuperantia [27], the RNA-binding proteins Pumillio and Nanos [28], the ESCRT-II complex member VPS36 [29] and and the RNA-binding protein/motor protein Dynein-mediator Eglitarian [30]. Of note, most of these interactions are based on binding of these proteins to the long 3’UTR of the *bcd* mRNA. This long stretch also harbors several distinct intrinsic functions such as the dimerization domain [25, 31], a mRNA stability region [32] and dependence of the translation efficiency of the mRNA, dependent on the length of the poly A tail [33, 34].

On the protein side, the most prominent targets of Bcd are the mRNA of the transcription factor Caudal that controls posterior development [35, 36], as well as the many transcription factors that control segmentation including the gap genes, pair-rule genes and segment polarity genes (reviewed by [37, 38]. Notably, Bcd also interacts with many other proteins and may not necessarily be involved in DNA-binding-mediated gene regulation [39]. Indirect evidence for Bcd being trapped in an actin network came from Bcd-movement studies showing strong assembly of Bcd in energids, an actin-rich zone [13, 40]. Whether this co-localization is due to direct binding to actin or just loose assembly in a protective actin shell to prevent degradation is currently unclear.

In this paper, we sought to define additional factors involved in shaping the *bcd* gradient. Through a series of novel approaches to analyze *bcd* gradient formation, we provide evidence that Bcd gradient formation is far more complex than previously thought and demonstrate that the gradient can be modulated in space and time.

## Results

### Wispy and PAP2, two poly A polymerases acting on *bcd* mRNA localization

In search of factors that can modulate the formation of the Bcd gradient, we stumbled over the *PAP2* and *wispy* (*wisp*) genes, the poly A polymerase orthologues in *Drosophila*, respectively [41–43]. PAP2 is the canonical poly A polymerase and is mainly required for poly A tail synthesis of the immature mRNA in the nucleus. Wisp, on the other hand, belongs to the family of atypical poly A polymerases of the GLD-2 family which was first identified in *C. elegans* [44] and subsequently later in most phyla [45, 46]. All GLD-2 poly A polymerase were shown to exert more specific functions than PAP2 and thus can be considered specialized poly A polymerases [46].

**Fig. 1:**
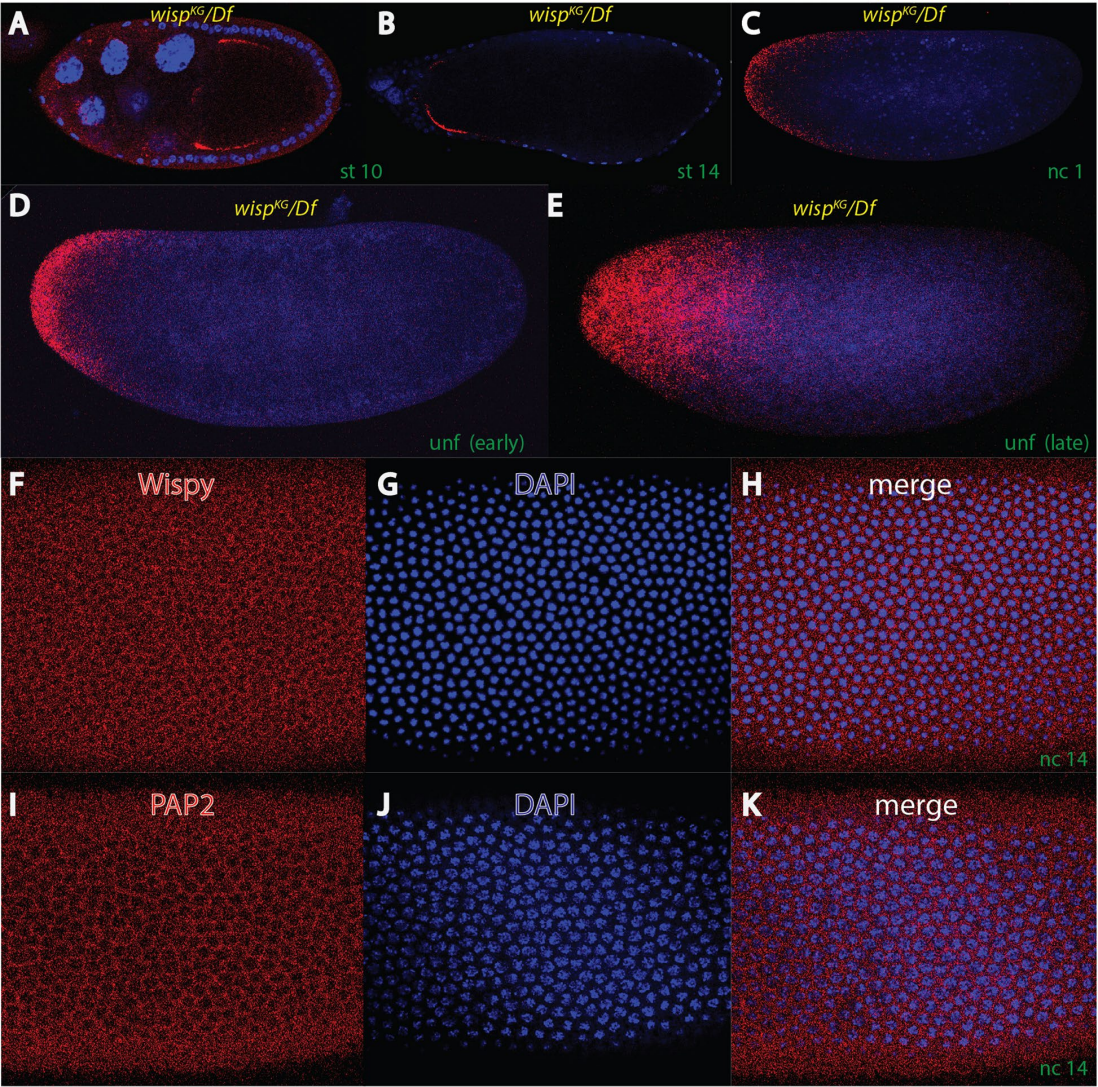
*bcd* RNA distribution in *wispy* mutants and expression of Wispy and PAP2 in embryos. **A**-**E** in situ hybridization of *wisp*^*GK05287*^/*Df(1)RA47* oocytes (**A**-**B**) and embryos (**C**-**E**) using *bcd* probes (in red) along with DAPI (in blue) to reveal nuclei. All panels are mid-sagittal single confocal sections mounted anterior end to the left and dorsal side up, where possible. In early staged oocytes, *bcd* mRNA is not transported to the anterior rim, rather it stays laterally in a stage 10 (**A**) or stage 14 oocyte (**B**). Consequently, in a *wisp*^*GK0287*^/*Df(1)RA47* embryo where growth is arrested during the first nuclear cycle (**C**), a deep cortical mRNA gradient is observed. In a freshly unfertilized *wisp*^*GK0287*^/*Df(1)RA47* embryo (**D**), a similar steep cortical gradient is observed as in (**C**), while in an old unfertilized *wisp*^*GK0287*^/*Df(1)RA47* embryo, the mRNA appears dispersed all over the anterior half. **F**-**K** single confocal sections on the apical surface of nuclear cycle 14 embryos, stained with anti-Wispy antibodies (**F**), DAPI staining (**G**) and the merge of both (**H**), anti-PAP2 antibodies (**I**), DAPI staining (**J**) and the merge of both (**K**). Note that both poly A polymerases stain the cytoplasm and are excluded from the nuclei

We decided to investigate the role of both poly A polymerases for *bcd* gradient formation, prompted by reports that both genes are involved in shaping the length of the poly A tail of the *bcd* mRNA during development [41, 43]. We first focused on the effect of poly A polymerases on *bcd* mRNA localization during oogenesis and early embryogenesis (Fig. 1). While in PAP2 mutants, by using a strong *hrg*^*PAP12*^ allele [41] showed the *bcd* mRNA localization pattern was indistinguishable from that of wild-type during oogenesis and embryogenesis (data not shown), oocytes from *wisp*^*KG/Df*^ mutant mothers, a *null* mutant, showed insufficient transport of the *bcd* mRNA to the anterior pole (Fig. 1A). Instead, the mRNA became localized laterally. Subsequently, in later staged oocytes, this lateral localization remained accentuated (Fig. 1B). As a consequence, in *wisp*^*KG/Df*^ embryos which arrest after completion of meiosis upon fertilization [42, 47], the *bcd* mRNA appeared in a broad anterior gradient (Fig. 1C), reminiscent of the final mRNA gradient at nc 14 [7, 11]. Interestingly, the *bcd* mRNA in *wisp*^*KG/Df*^ mutants always remained at the cortex and was never transported into the interior of the egg nor of the oocyte, suggesting an intact non-permissive territory for *bcd* mRNA transport [13].

When unfertilized *wisp*^*KG/Df*^ embryos were analyzed, we made interesting observations regarding the *bcd* mRNA pattern: freshly laid embryos showed a broad anterior *bcd* mRNA cap (Fig. 1D), as in fertilized and arrested *wisp*^*KG/Df*^ embryos, however, if unfertilized eggs were fixed 3 h after egg laying, the mRNA pattern was no longer retained to the cortex, but showed uniform movement into the egg (1E). A similar observation of an interiorwards movement of the *bcd* mRNA in unfertilized (wild-type) eggs was observed in the past [11], however, there is a distinct difference: in wild-type eggs, the interiorwards movement of the *bcd* mRNA followed a defined path, ascribed to the existence of a defined interior microtubular transport system, while in *wisp*^*KG/Df*^ unfertilized embryos, movement of the *bcd* mRNA appeared randomly throughout the interior of the egg. This observation suggests that proper polyadenylation is crucial for establishing the cytoarchitecture of the egg, consistent with the observation that *wisp*^*KG/Df*^ embryos stop development at nc 1 at the moment when meiosis is completed [42, 47].

To analyze the spatial pattern of both poly A polymerases, we employed specific antibodies against each protein [41, 47]. We used a fixation method that allowed high sensitivity combined with low background caused by low autofluorescence from the egg [13, 21, 48]. Interestingly, both Wispy and PAP2 proteins showed cytoplasmic localization and were excluded from the nucleus at nc 14 (Fig. 1F-K), consistent with data on Wisp localization [26], however in conflict to the reported nuclear localization of PAP2 at that stage [41].

### Altering the Bcd gradient through modulation of Wispy

**Fig. 2:**
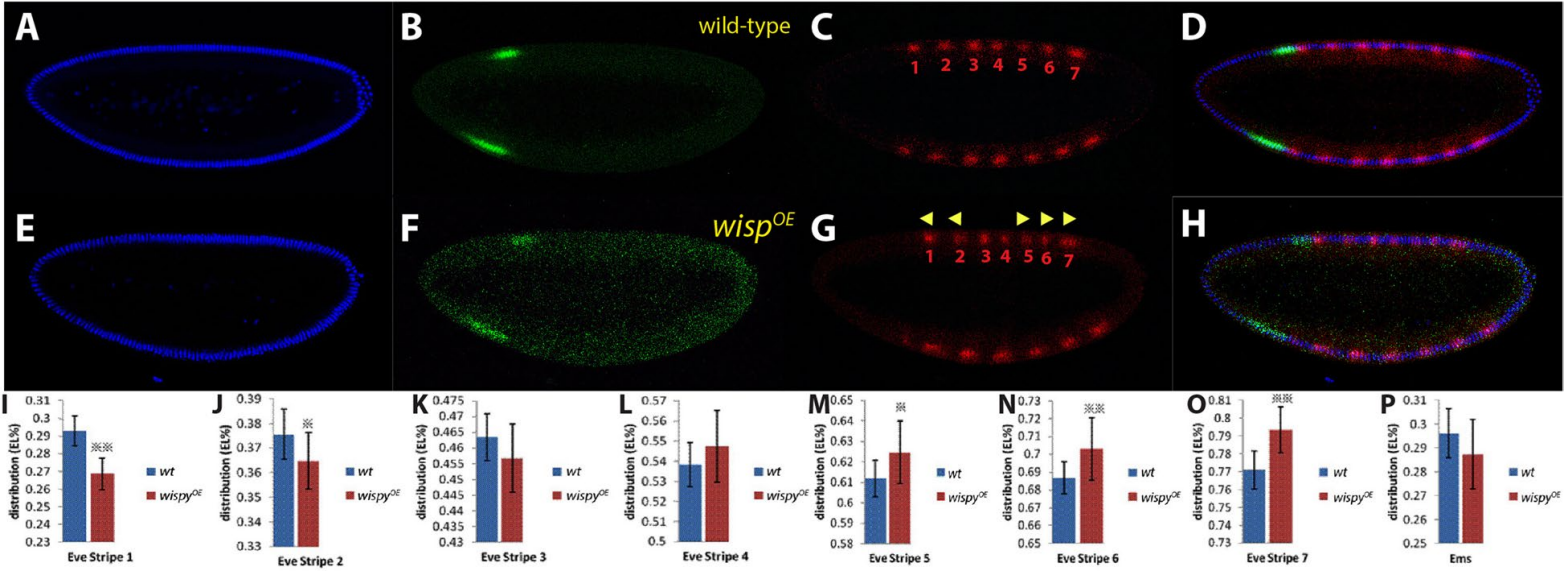
Influence of *wispy* over-expression (*wisp*^*OE*^) on the fate of the segmental anlagen. **A**-**D** single mid-sagittal confocal section of a nuclear cycle 14 wild-embryo stained for DAPI (**A**), Ems (**B**), Eve (**C**) and the merge of (**A**-**C**) in (**D**). **E**–**H** single mid-sagittal confocal section of a nuclear cycle 14 *wisp*^*OE*^ embryo stained for DAPI (**A**), Ems (**B**), Eve (**C**) and the merge of (**A**-**C**) in (**D**). Eve stripe numbers are indicated in red in (**C** and **G**). Triangles ◀ in (**G**) indicate shift of expression towards the anterior. Triangles ► in (**G**) indicate shift of expression to the posterior. **I**-**O** statistic analysis of the positions of the anterior boundaries of the individual Eve stripes 1–7 in wild-type embryos (blue bars) and *wisp*^*OE*^ embryos (red bars). **P** statistic analysis of the positions of the posterior boundaries of the Ems expression domain in in wild-type embryos (blue bars) and *wisp*^*OE*^ embryos (red bars). ※ 0.05 < *P* < 0.01; ※※ *P* < 0.01. The error bars indicate standard deviations. Tables 1 and 2 summarize the findings

Since it was known that the poly A tail length of the *bcd* mRNA changes with time and alters the translation efficiency of the Bcd protein [33, 34], it was conceivable to investigate if modulation of the activities of the two poly A polymerases would also alter the Bcd gradient. Overexpression of PAP2 was shown to increase the poly A tail of *bcd* mRNA during oogenesis as well as in embryogenesis, but conferred early embryonic lethality during the first nuclear cycles [41]. Hence, analysis on the effect on the *bcd* gradient during later nuclear cycles was impossible. *wisp* over-expression during the germ-line, however, did not confer early embryonic lethality, nor did it affect growth and morphogenesis of the oocyte, thus was suitable for testing the effect on *bcd* gradient formation. To evaluate the extent how the Bcd gradient is altered at *Drosophila* blastoderm, we chose a gap gene, *empty spiracle* (*ems*) and a pair-rule gene *even-skipped* (*eve*), respectively, as read-out systems. These genes were shown to readily respond to changes of the Bcd gradient, by shifting their expression domains along the anterior–posterior (A-P) axis [49–51]. In wild-type embryos, Ems was expressed in an anterior circumferential stripe with its anterior margin located around 30% egg length (EL, 0% is anterior, 100% is posterior; Fig. 2B). Eve, on the other hand, was expressed in seven regularly-spaced stripes starting from around 30% to 80% EL (Fig. 2C).

**Table 1:** Shift of Eve stripes in wild-type and *wisp*^*OE*^ mutants

	1^a^	2^a^	3^a^	4^a^	5^a^	6^a^	7^a^	T(^o^ C)	n^b^
wild type	29.30% ± 0.85%	37.56% ± 1.02%	46.35% ± 0.74%	53.83% ± 0.90%	61.19% ± 0.90%	68.69% ± 1.03%	77.09% ± 1.10%	29	16
*wispy*^*OE*^*(I)*	26.87%** ± 0.88%	36.47%* ± 1.15%	45.68% ± 1.09%	54.74% ± 1.78%	62.47%* ± 1.52%	70.29%** ± 1.76%	79.35%** ± 1.28%	29	13

0–4 h embryos from wild-type and *wispy*^*OE*^ mutants were collected at 29° C, respectively. Embryos were stained with an antibody against Eve. Eve stripes were measured by ImageJ. Data were analyzed by using student t-test (*p*-value of 0.05) and reported as means ± SE

^a^Eve stripes

^b^number of embryos examined

*OE* Overexpression

^*^0.05 < *P* < 0.01; ** *P* < 0.01

**Table 2:** Shift of the Ems band in wild-type and *wisp*^*OE*^ mutants

		T(^o^ C)	n^b^
wild type	29.61% ± 1.06%	29	16
*wispy*^*OE*^*(I)*	28.74% ± 1.45%	29	13

0–4 h embryos from wild-type and *wispy*^*OE*^ mutants were collected at 29° C, respectively. Embryos were stained with an antibody against Ems. Ems band was measured by ImageJ. Data were analyzed by using student t-test (p-value of 0.05) and reported as means ± SE

^b^number of embryos examined

*OE* Overexpression

If *wisp* was over-expressed (*wisp*^*OE*^), then statistically-significant shifts of the Eve stripes were observed: stripes 1 and 2 were shifted anteriorly (Fig. 2G, I, J), stripes 3 and 4 remained virtually unchanged (Fig. 2G, K, L), while stripes 5–7 showed a posterior wards shift (Fig. 2G, M, N, O). The gap gene EMS, however, did not change significantly (Fig. 2G, P), but nevertheless followed the trend of Eve stripes 1 and 2. Since anteriorwards shifts of Eve stripes is a sign of decreased Bcd activity, posteriorwards shifts points towards an increased Bcd activity [3]. An combined interpretation of the shifts led to the conclusion that *wisp*^*OE*^ had a deteriorating effect on the Bcd gradient in the anterior part of the embryo, while it appeared attenuated in the posterior part. This would suggest that the exponential curve of Bcd in wild-type embryos following mathematical rules [7, 52] would be flatter in the anterior part, would exhibit "normal" levels in the middle, but would show higher levels in the posterior part in *wisp*^*OE*^. It follows that *wisp*^*OE*^ will likely lead to a change in the segmental anlagen at blastoderm stage.

**Fig. 3:**
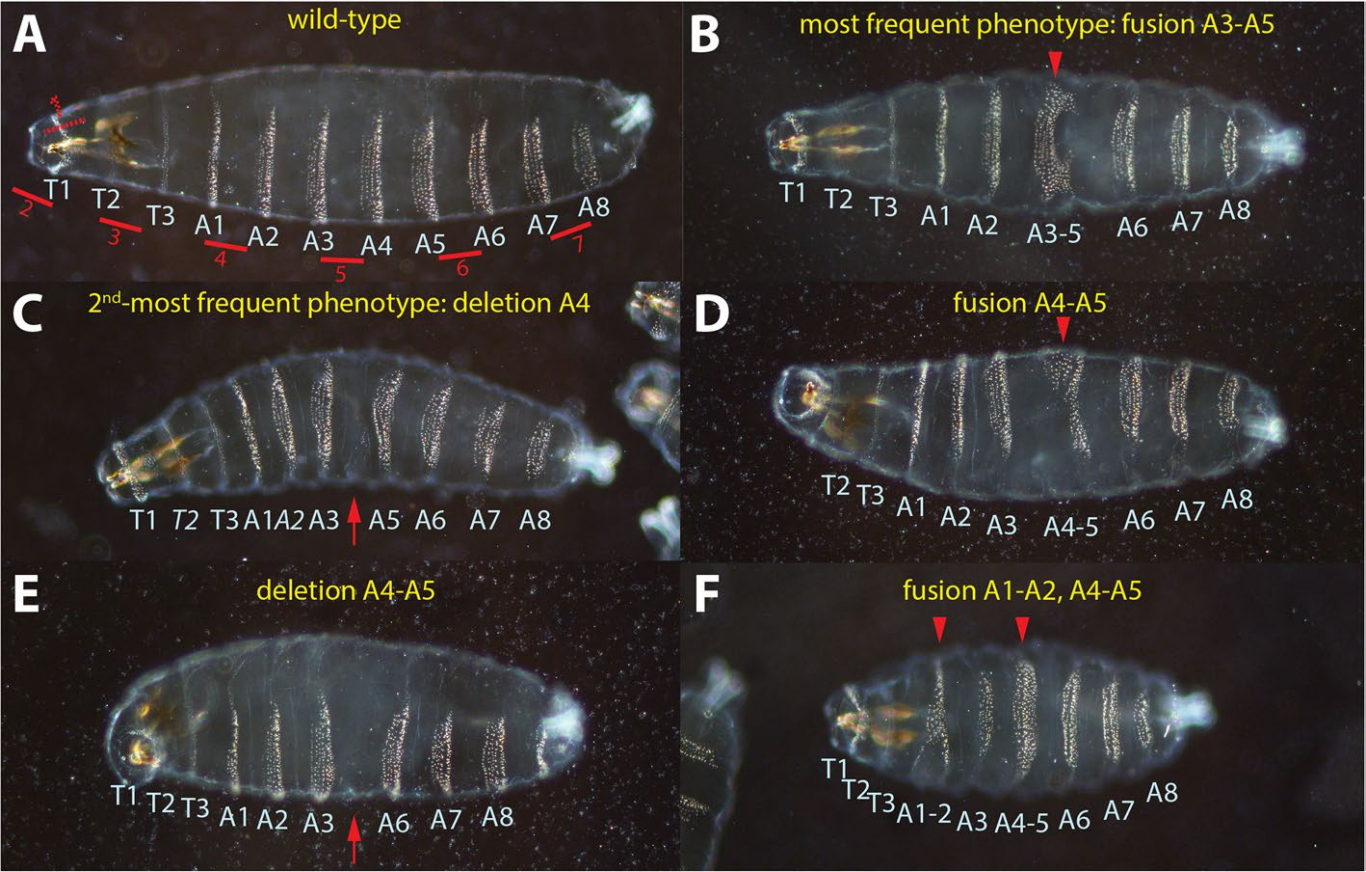
Cuticle analysis of *wispy* over-expression (*wisp*^*OE*^). **A** cuticle of a wild-type larva serving as control. The larval body contains three thoracic (T1-T3) and eight abdominal (A1-A8) segments. The location of the Eve stripes at blastoderm stage are drawn and numbered in red with respect to their later position. Stripe 1 will affect internal structures and is drawn dashed. **B** embryo from a *wisp*^*OE*^ cross showing the most-frequent phenotype as fusion of A3-A5. **C** embryo from a *wisp*^*OE*^ cross showing the 2^nd^-most-frequent phenotype as a deletion of A4. **D**-**F** less-frequent phenotypes observed in embryos a *wisp*^*OE*^ cross showing fusion of A4 and A5 (**D**), deletion of both A4 and A5 (**E**) and pairwise fusions of A1 and A2 as well as A4 and A5. Segmental fusions are indicated by red arrowheads, segmental deletions by red arrows

To corroborate the possibility of a shift of the anlagen at the blastoderm stage, we reasoned to analyze the cuticle pattern upon *wisp*^*OE*^. Several classes of cuticle phenotypes were observed, all centered around abdominal segments 3 to 5 (A3-5) with a "hotspot" at A4. The most frequent phenotype was a fusion of A3-5 (Fig. 3B), while a minor fraction of embryos showed deletion of A4 (Fig. 3C), interpreted as a milder form of the phenotype associated with fusion of A3-5. Further phenotypes that were observed were: fusion of A4-5 (Fig. 3D) and, as a more pronounced effect, a deletion of A4-5 (Fig. 3E) and pairwise fusions of A1-2 and A4-5 (Fig. 3F). [53] showed that the Eve stripes corresponded to uneven-numbered parasegments. We could note that the region centered around A3-5 corresponded to Eve stripes 5 and 6, both of which were significantly shifted to the posterior upon *wisp*^*OE*^.

### Spatial differences of the poly A tail length along the A-P axis.

One of our working hypotheses for the altered Bicoid activity along the A-P axis was the question if the poly A tail length would change during the A-P transport of the *bcd* mRNA as part of a larger RNP to the posterior. This hypothesis was fueled since it was shown that the *bcd* mRNA particle consisted of several components, one of which turned out to be Wisp, because *bcd* mRNA could be co-immunoprecipitated with Wisp [26]. Hence, it seemed conceivable to assume that the poly A tail of *bcd* mRNA would be elongated while the mRNA migrated to the posterior. As a result, *bcd* mRNAs located more posteriorly would exhibit a longer poly A tail than those that are located more anteriorly. A further consequence of this scenario would be that posterior *bcd* mRNA molecules would possess a higher capacity to translate Bcd protein than anterior ones. Hence, this scenario would represent another level to control the establishment of the Bcd gradient, an aspect that was never considered in the past.

**Fig. 4:**
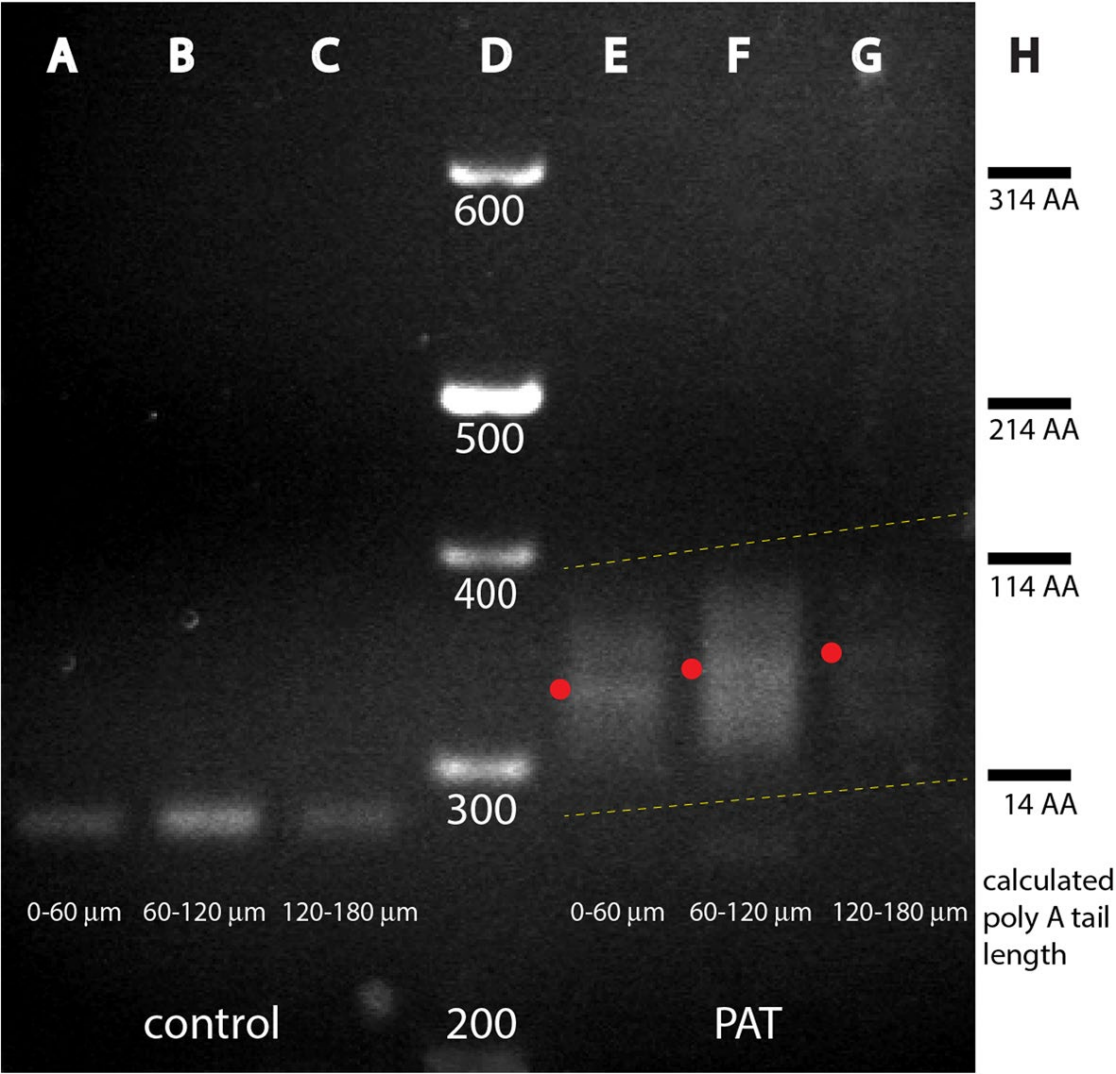
Spatial distribution of poly A tail length of *bcd* mRNA along the A-P axis of *Drosophila* using PAT analysis. Poly A tail (PAT) analysis of *bcd* mRNA isolated from 3 consecutive 60 μm sections (**A**, **B**, **C**; **E**, **F**, **G**) from ten nc 13 embryos starting from the anterior tip and cutting to the posterior as indicated at the bottom. **A**, **B**, **C** internal PCR primer control of *bcd* to evaluate abundance and quality of the isolated RNAs. **D** DNA size marker. **E**, **F**, **G** PAT analysis showing increased sizes of smears suggesting that the poly A tail lengths change with the location along the A-P axis. **H** calculated poly A tail length

To tackle this hypothesis, we decided to mount ten staged nc 13 embryos into an embedding medium ensuring that embryo orientation and their anterior ends were aligned to one line. Subsequently, 3 serial sections of 60 μm each were executed in the cryostat, followed by extraction of the RNA as described by [54]. The isolated RNA was then subjected to a PAT analysis, identical to that of [33] and the PCR products were fractionated on a 2.5% agarose gel (Fig. 4). Control lanes (lanes 4A-C) showed that the integrity of the RNA was maintained, although their concentration varied among the 3 samples. If assayed for *bcd* poly A tail length, the first 60 μm contained a smear with a prominent band at around 50 AAs (lane E). In the next 60 μm, the smear moved upwards, showing a prominent band at around 65 AAs (lane F), while the last 60 μm analyzed showed a continuation of the upwards trend with a minor peak at around 75 AAs (lane G).

These data suggested that the length of the poly A tail indeed showed spatial variation and that more posteriorly-located *bcd* mRNA particles possessed a longer poly A tail. This data points towards that Wisp may control the length of the poly A tail while being associated with the *bcd* RNP [26]. Moreover, the few posteriorly-located particles can translate Bcd stronger and thus may stretch the formation of the gradient to the posterior. We do not know, however, how long Wisp is associated the *bcd* RNP and whether the association is constant over the A-P range.

### Modulating the action of *bcd* in time

The Bcd gradient is established from the time of formation of the syncytial blastoderm and regulates the activity of gap genes and pair-rule genes during nc 11–14 [55]. While nature would ensure that the correct amount of Bcd protein would end up in every nucleus at nc 14, we pondered what would happen if the timely production of Bcd could be altered such that more than 14 nuclear cycles would be made available to the embryo, and what the consequences for the segmental anlagen at blastoderm would be. We expected that such a scenario would likely involve further transport of the *bcd* mRNA to the posterior and longer exposure of the mRNA to polyadenylation. To test this hypothesis, we searched for mutations that would enable extra nuclear cycles during the syncytial blastoderm stage. One of them, *maternal haploid* (*mh*) [56–60], a maternal effect mutation encoding a metalloprotease enriched at the largest satellite repeats seemed suitable to test this hypothesis. We used *mh*^*1*^, an allele that occasionally allows progression through a 15^th^ nc, but soon afterward confers embryonic lethality [56, 57].

**Fig. 5:**
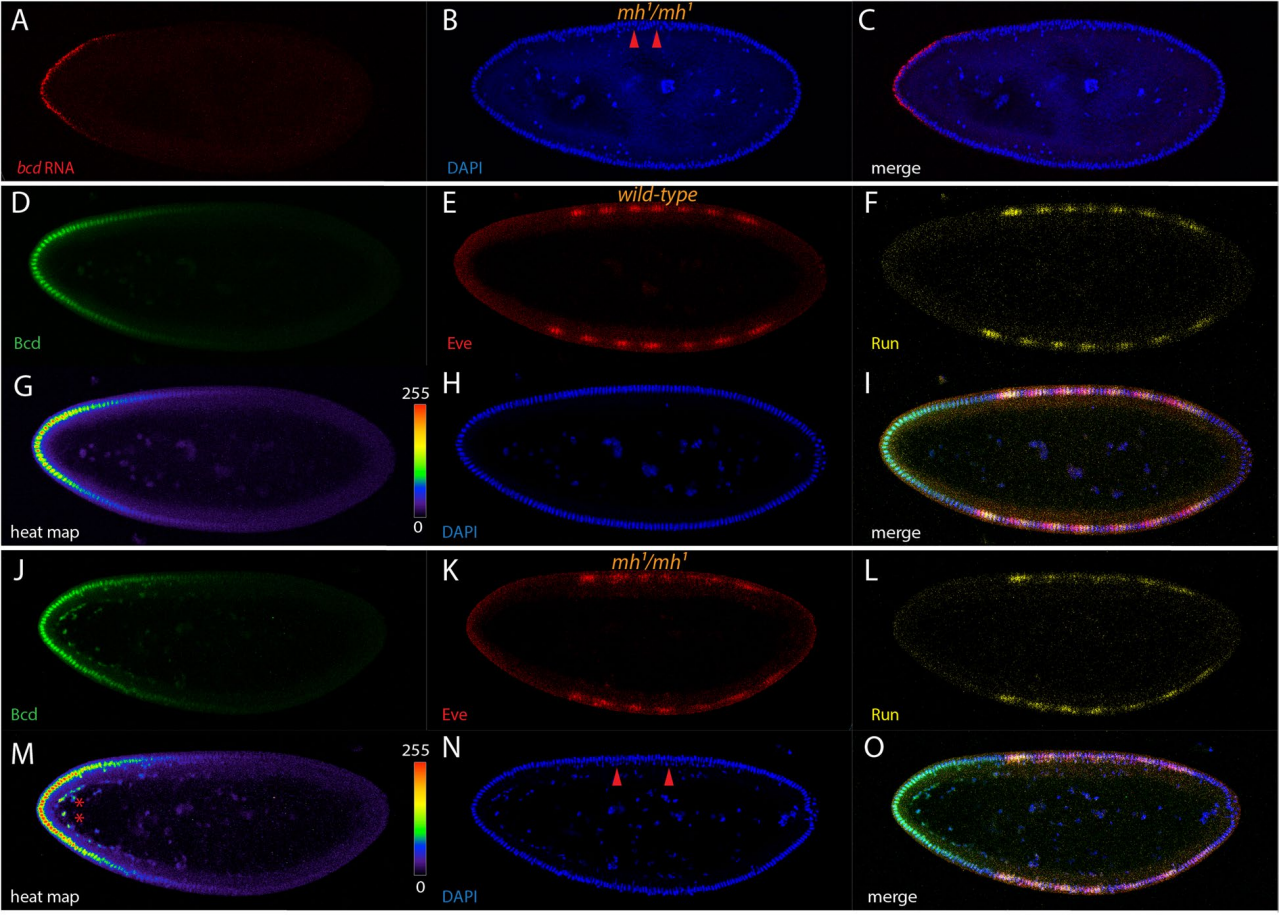
Modulation of the *bcd* gradient by an extra nuclear cycle. Pictures represent midsagittal confocal planes of embryos oriented with their dorsal side up and anterior to the left. **A**-**C** a nc 15 embryo from *mh*^*1*^/*mh*^*1*^ mothers stained for *bcd* mRNA(A, red), DAPI (**B**, blue) and the merge of (**A**-**B**) in (**C**). Note the double layer of nuclei due to an extra round of nuclear division, arrowheads in (**B** and **N**). **D**-**I** a nc 14 wild-type embryo stained for Bcd protein (**D**, green), Eve (**F**, red), Run (**G**, yellow), heat-map of the intensities of Bcd protein concentrations including scale from 0–255 (**G**), DAPI (**H**, blue) and the merge of (**D**-**F**, **H**) in (**I**). **J**-**O** nc 15 embryo from *mh*^*1*^/*mh*^*1*^ mothers stained for Bcd protein (**J**, green), Eve (**K**, red), Run (**L**, yellow), heat-map of the intensities of Bcd protein concentrations including scale from 0–255 (**M**), DAPI (**N**, blue) and the merge of (**J**-**L**, **N**) in (**O**)

We first checked how the *bcd* mRNA gradient would be affected in embryos from *mh*^*1*^/*mh*^*1*^ mothers. Around 10% of *mh*^*1*^/*mh*^*1*^ embryo reached the cellular blastoderm stage and exhibited a higher number of blastoderm nuclei due to an extra round of nuclear division, hence are at nc 15. Since the number of nuclei at cellular blastoderm, usually ∼6000 nuclei in wild-type embryos, was limited due to space constraints, many of these extra nuclei appeared now displaced towards the yolk (Fig. 5B, arrowheads). As far as the mRNA gradient was concerned, in nc 15 *mh*^*1*^/*mh*^*1*^ embryos, the *bcd* mRNA gradient appeared indistinguishable from that of wild-type nc 14 embryos (Fig. 5A, C). Basal to apical transport of the mRNA, normally occurring during the first 15 min of nc 14 in wild-type embryos [7], occurred in nc 15, instead (data not shown). This data suggests that the formation of the *bcd* mRNA gradient appears independent of the number of nuclear cycles.

We then focused on the appearance of the Bcd protein and used a set of pair-rule genes, Even-skipped and Runt, to monitor the appearance of downstream targets in relation to the Bcd gradient by using triple antibody staining. Moreover, we highlighted the appearance of the Bcd gradient using a heat-map analysis to eventually detect subtle changes in the gradient [7, 13].

In wild-type nc 14 embryos, Bcd formed an impressive gradient (Fig. 5D, G) and the two pair-rule genes, Eve (Fig. 5E) and Runt (Fig. 5F) appeared as 7 stripes, respectively, with distinct relative phasing to each other (Fig. 5I). In nc 15 *mh*^*1*^/*mh*^*1*^ embryos, it could be noted that the Bcd gradient appeared indistinguishable from that in wild-type embryos (Fig. 5J, M), as did the mRNA analysis (Fig. 5A, C). However, we could note that the surplus of internalized nuclei at the anterior side were strongly filled with Bcd (Fig. 5J, M) suggesting that more Bcd was produced than in a comparable nc14 wild-type embryo (Fig. 5D, G). Of note, the gradient appeared normal (Fig. 5M), compared to wild-type (Fig. 5G). Regarding the fate map of the segmental anlagen, no changes were apparent, as the relative positions of both Eve (Fig. 5K) and Run (Fig. 5L) stripes along the A-P axis appeared unchanged, compared to the respective wild-type situation (Fig. 5E, F). From this data, we could conclude that altering the timely action of *bcd* did not lead to a change in the intrinsic properties of the gradient.

### Genetic manipulation of Bcd movement

Recent studies showed that the movement of Bcd strongly depended on an intact cytoarchitecture of the early *Drosophila* egg [1, 13]. In those studies, it became evident that Bcd never moved into the inner core of the egg, but rather stayed at and moved along the cortex. Hence, the inner core defined a non-permissive territory [13], in agreement with the proposed mechanisms associated with the ARTS model [7]. However, [13] could demonstrate that the properties of inner core could be altered, upon administration of small molecules against microtubules that compromise its cytoarchitecture, such that the core became permissive. As a consequence, in drug-administered eggs, Bcd moved along a broad front into the egg and thus appeared to conform the SDD model [13].

**Fig. 6:**
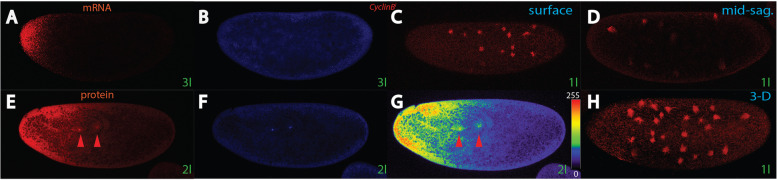
Modulation of the Bcd gradient by *Cyclin B*. Pictures represent midsagittal confocal planes of embryos oriented with their dorsal side up and anterior to the left, except (**C**, **H**). **A**, **B** a nc 3 *CycB* RNA^i^ embryo stained for *bcd* mRNA (**A**) and DAPI (**B**). **C**, **D**, **H** a nc 1 *CycB* RNA^i^ embryo stained with mab YL_1,2_ detecting freshly-made tubulin, and analyzed as a surface confocal Z-stack section (**C**) and a mid-sagittal confocal Z-stack of the same embryo (**D**), as well as a 3-D reconstruction combining multiple Z-stacks of the same embryo in (**C**, **D**) to reveal the microtubular pattern in the outermost 30 μm of the cortex. **E**, **F** mid-sagittal nc 2 *CycB* RNA^i^ embryo stained with Bcd antibody. Arrowhead denote filling of nuclei with Bcd. **G** heat-map of the intensities of Bcd protein concentrations in embryo (**E**) including scale from 0–255. Arrowhead denote filling of nuclei with Bcd. **H** surface 3-D reconstruction of the confocal stack used in (**C**, **D**) to demonstrate extensive formation of spontaneous asters, despite lack of MTOCs at the cortex. Stages of embryos are denoted in green and follow the nomenclature of [12]

We then wondered if we could identify genes that upon inactivation would allow movement of Bcd into the inner core of the embryo instead. Among the many genes tested, one gene, *Cyclin B*, (*CycB*) [61–63], when knocked-down, showed the desired effect (Fig. 6). *Cyclin B* is involved in regulation of nuclear cycles, in particular the entry point into mitosis in conjunction with *Cdk1* [64], as well as in controlling the length of microtubules [63].

We first analyzed the behavior of *bcd* mRNA in *CycB* mutants using *RNA*^*i*^ lines in combination with a maternal driver, termed *CycB*^*i*^. *CycB*^*i*^ mother did not produce many eggs due to fact that oogenesis was compromised. Moreover, *CycB*^*i*^ embryos were smaller in size and showed early embryonic lethality with halted development during the first four nuclear cycles, consistent with observations from [62]. The *bcd* mRNA pattern did not show any marked change compared to wild-type and the mRNA stayed at the anterior pole, although the anterior cap did not show the compacted form as in wild-type embryos Fig. 6A; [7, 65]. Due to the fact that *CycB* also controls the length of microtubules [62], we analyzed the behavior of MTs using YL_1,2_, a monoclonal antibody previously shown to detect the MT network that is responsible for transporting the *bcd* mRNA to the posterior [7, 11]. In *CycB*^*i*^ embryos, many cortical (Fig. 6C), but also internal (Fig. 6D) MT asters were detected, similar to the ones detected in *αTub67C*^*3*^ embryos [11]. Of note, none of these asters were associated with a MTOC in vicinity of nuclei, hence represented autonomous aggregations of MTs into asters. Moreover, the number of cortical MT threads was markedly increased. By combining a whole Z-stack to obtain a 3-D picture, many random MT asters and a dense MT network could be detected that were not associated with nuclei (Fig. 6H). Thus, in *CycB*^*i*^ mutants, the MT pattern appears random and *CycB*^+^ appears to suppress these asters. Possibly, due to absence of any mitotic cycles and its major regulator (*CycB*), a surplus of free MT monomers is available to enable a self-organizing process creating these asters.

Next, we analyzed the Bcd protein pattern in *CycB*^*i*^ embryos. In contrast to the mRNA (Fig. 6A), the protein showed a complete different pattern (Fig. 6E, G). Instead of moving along the cortex in a coordinated manner with time [13], a portion of the protein moved to the interior in a broad front, best seen in the heat-map analysis (Fig. 6G). This type of movement is highly reminiscent in embryos that were treated with the MT-degrading drug Vinblastine [13], thus appeared to conform the SDD model. Moreover, internal nuclei were filled with Bcd protein suggesting that the changed cytoarchitecture of the non-permissive territory of the inner core enabled movement of Bcd deeply into the interior.

## Discussion

We have shown that several parameters have a profound effect on the formation and appearance of the *bicoid* gradient, as well as movement of the Bicoid protein in the *Drosophila* embryo. The first parameter concerned the translation efficiency of *bcd* mRNA and consequences for the Bcd gradient and its intrinsic information. The second one pertained to the duration where *bcd* was active and how this affected the position of the segmental anlagen. The third one applied to a genetic influence on the cytoarchitecture that ultimately led to a completely different Bcd movement.

Physical interaction studies using microarray analysis involving the Wisp poly A polymerase as bait revealed that Wisp interacted with a plethora of mRNAs, among those *bcd* mRNA was also detected [26]. Since *bcd* mRNA is incorporated in a larger RNP which contains many other proteins such as Staufen [25], it appeared conceivable to assume that Wisp, for a certain time interval was physically associated with the RNP complex. Since Wisp localization was cytoplasmic (Fig. 1F, [43], a possible scenario could be that Wisp would have access to the RNP for poly A tail modification of the *bcd* mRNA, but only when the RNP transport along MTs was halted. This scenario would be consistent with the proposed mechanism where the mRNA is transported when the MTs are built up. However, since the MT network for transport was only built up during Metaphase and early Anaphase, while being non-existent during the remaining phases of a nuclear cycles, it could transport the mRNA during a short time interval of a nuclear cycle. Thus, Wisp would have the majority of a nuclear cycle at its disposition to act on *bcd* mRNA because the RNP is not moving. Another scenario would be that Wisp is integral part of the RNP and moves with it to the posterior.

In the past, efforts were made to investigate on intrinsic functions of either the *bcd* mRNA or the Bcd protein that would play a role in gradient formation. On the mRNA side, a motif immediately downstream of the stop codon was shown to mediate stability of the mRNA [32], or a nonamer sequence further downstream that was shown to be important for its localization [66], along with studies that revealed the binding sites on the *bcd* mRNA for the RNA-binding protein Staufen [25]. Furthermore, it was shown that *bcd* mRNA always assembled as a dimer within the RNP and the assembly could be attributed to a *cis*-element responsible for dimerization that is localized in a stem-loop domain (domain III), [31]. Now, in this report, we added another tool to shape the gradient via an intrinsic function: the poly A tail length function controlling the translation efficiency, combined with spatial differences of the poly A tail length along the A-P axis (Fig. 4). We wish to propose this mechanism to raise the initial positional information defined by the mRNA gradient to the next level, with help of differential translation efficiency of the mRNA along the A-P axis. From previous studies, it became evident that the mRNA gradient extended far to the posterior, at least to 40% EL [7, 8, 11], and may extend even further to the posterior, currently hampered by restrictions in the sensitivity of the RNA in situ detection technique. Hence, the few *bcd* RNPs that could be transported to the far posterior most likely contribute with a higher efficiency to locally translate Bcd protein and thus are able to modify the pre-pattern mRNA gradient.

Recently, it was demonstrated that the translation efficiency of ribosomes of the early *Drosophila* egg is not linear along the A-P axis, exemplified by an analysis on the gap gene *hunchback* (*hb*) [67]. A completely new hallmark of this report was that *hb* expression levels would not only be dependent on the binding of the necessary transcription factors driving the gene, e.g. by *bcd*, but also at the level of translation. It was then proposed that translation of *hunchback* would be controlled by other *trans*-acting factors and that this regulated *hunchback* translation would constitute another tool to shape the expression of *hb* along the A-P axis. Likewise, we wish to propose that the translation of *bcd* could be controlled not only by its intrinsic properties, such as the length of its poly A tail, but also by *trans*-acting factors such as miRNAs, known to regulate translation of protein across most phyla [68]. Consistent with this observation, the 3’UTR of *bcd* contains a highly-conserved motif for binding to *miR305* (FlyBase; [69]) immediately upstream of the stability motif [32]. Moreover, *miRNA 305* was shown to be one of the major inhibitor for Bcd translation in the oocyte [70] through binding of *miRNA 305* to this highly-conserved motif.

Our studies demonstrated that manipulating the time when Bicoid is active and for how long it can act did not have a profound effect on the regulation of the cascade of segmentation genes (Fig. 5). While the result was rather surprising, it suggests that possibly the segmental anlagen are defined already at nc 14, likely through Bcd, and that this definition is locked afterwards. An alternative interpretation could be that the definition of the segmental anlagen is also depended on other determining factors, and thus not solely dependent on Bcd.

### Increased sensitivity of abdominal segments A4 and A5 upon perturbations

Manipulation of the Bcd gradient in Fig. 2 revealed that the changes were not linear, e.g. a linear posteriorward shift of the Eve stripes as observed in *Drosophila* mutants that provide a higher Bcd dose [13, 71]. Rather, the anterior two stripes moved to the anterior and the posterior three stripes moved to the posterior. Stripes 3 and 4 remained unchanged and thus represented a fixed point where the exponential Bcd intensity curve [7, 52] was turned slightly counter-clockwise at the position of Eve stripes 3 and 4 to define a new decay curve. Anterior to this position, the curve was lowered, posterior to this position, the curve appeared elevated. Consistent with this proposal, the cuticle region corresponding to Eve stripes 3 and 4, T2/T3 and A1/A2, respectively appeared mostly unaffected (Fig. 3). This raises the critical question why the region of A3 to A5, corresponding to parasegments 9–11 is particularly sensitive to changes in the cuticle. One explanation could be that the over-expressing driver line dumps GAL4 protein non-uniformly into the egg, or the protein translation properties of this region are not identical to that of the rest of the embryo.

### The problem with the rescue of the *bcd* phenotype.

In the past, many reports validated their transgenic *bcd* constructs by analyzing the phenotypic consequences or the behavior of e. g. a Bcd-GFP fusion in a *bcd*^*−*^ background by scoring the larval cuticle for full rescue [4, 6, 72–81]. We wish to remind the community that this is an easy way out to “validate” a *bcd* construct, but does not fully reflect the biological function of Bcd for the patterning process. This notion is based on the following two arguments: 1) even under conditions where the segmental anlagen are shifted in one direction, e. g. to the posterior, through increased doses of Bcd, the resulting larval pattern is normal. This apparent conundrum resolves because correction mechanisms during later stages are in play that restore the faulty programming of the segmental anlagen back to wild-type [13, 77, 82]. 2) The *bcd*^*E1*^ allele [2] that was used in all cases to provide a *bcd*-null background is not a true *bcd* null allele, as it only removes the activity of the large *bcd* isoforms. Complete removal of the *bcd* locus has a much stronger embryonic phenotype than any strong *bcd* allele, e.g. *bcd*^*E1*^ (Baumgartner, manuscript), which makes claims on any role of *bcd* subject to another through revision.

### Bcd movement in *CycB* mutants appears to fit the SDD model

In the past, the SDD model has been the predominant model to explain the occurrence of the Bcd gradient [3, 5, 6]. The model also proposed that Bcd would diffuse uniformly throughout the egg, fueled by simulation studies using fluorescent Dextran particles [83] which indeed showed a broad inwards movement of the particles and thus appears to fit the SDD model. Unfortunately, in these studies, it was never taken into consideration that the composition of the egg may not be uniform, but rather would contain territories shown to have a profound effect on Bcd movement [1, 13]. The two territories, the cortex and the inner core were already discovered several decades ago [12, 84], but were never discussed in conjunction of Bcd movement.

*CycB* is the first identified genetic component that had a profound effect on Bcd movement. When compromised, Bcd was allowed to migrate into the interior non-permissive territory (Fig. 6) and thus appeared to conform the SDD model. *CycB* is a regulator of microtubule activity and length, however, the gene is dispensable for mitosis and embryonic viability [85]. Absence of *CycB* affected oocyte growth, leading to reduced yield in eggs [62]. At the molecular level, lowering the doses of *CycB* increased the occurrence of microtubular asters, often resulting in thick and long microtubules and increased numbers of asters without nuclei in the center [62], in agreement with our *CycB*^*i*^ analysis (Fig. 6H). Possibly, the pool of available Tubulin monomers is affected which ultimately may weaken the Tubulin cytoarchitecture in the interior of the egg and thus allows Bcd to penetrate the inner yolk. Similar formation of spontaneous asters were detected in a weak *αTub67C* mutation [11], however, no data were made available regarding Bcd movement. We can conclude that *CycB* is a regulator of the cytoarchitecture of the non-permissive territory located in the interior, most likely by weakening the microtubular integrity of this territory.

## Conclusion

We have shown, through a number of experiments that the formation and appearance of the Bcd gradient is probably far more complex than previously thought and that this process involves a plethora of factors that help to shape the gradient. We demonstrated that the Bcd gradient formation is based on *trans*-acting factors that were previously not considered to exist. Hence, this data may open our view and may lay the foundation for further discoveries how Bcd gradient formation is governed.

## Materials and methods

### *Drosophila* stocks and genetics

Canton-S stock from Bloomington (No. 64349) was used as control. The *wispy* overexpression (*wisp*^*OE*^) stocks were generated by cloning a wispy full-length cDNA (based on RE03648, kindly provided by M. Simonelig) into a modified pUASP vector (kindly provided by M. Simonelig) where the *K10* 3'UTR was replaced by the SV40 3'UTR. This step is absolutely essential, as the *K10* 3'UTR appears to be regulated by *wispy* (M. Simonelig, pers. comm.). Standard germline transformations were performed by BestGene (USA). The *wispy* mutant in Fig. 1 was *wisp*^*KG05287*^ (Bloomington stock # 16,467) and was used in combination with the deficiency *Df(1)RA47* (Bloomington stock # 961), as described in [43]. The *y*^*1*^* w*^*a*^* mh*^*1*^*/FM7a* stock was from Bloomington, stock number 7130. The *CycB* RNA^i^ stock was *P[TriP.CycB*^*HMS01871*^*]attP2*, Bloomington stock number 38957. The 2^nd^-chromosome maternal *GAL4*-driver line *V32* was obtained from the Perrimon lab and was used for all over-expression and *RNA*^*i*^ experiments at 29°. Flies were fed with standard fly food (Bloomington recipe) and were exposed at 29^°^ C to boost the efficacy of the maternal GAL4 driver.

### Cuticle preparations

Embryos were collected in 24 h. interval, incubated > 36 h., dechorionated in 50% bleaching solution, fixed in 25% formaldehyde for > 5 h., devitellinized, mounted in Hoyer’s medium and incubated at 65^°^ C for 3–5 days, as described [13].

### Antibody staining and fluorescent in situ hybridization

Embryos were heat-fixed for immunostaining. Rabbit antibodies against wispy and PAP2 were obtained from M. Wolfner and M. Simonelig, respectively, and were both used at 1:1000. The monoclonal antibody against Eve (DSHB) was used at 1:250. Rabbit-anti-Bcd antibodies [11] were used at 1:1000, Guinea pig antibodies against Run were obtained from Kuni Saito and were used at 1:1000, rabbit-anti-Ems antibodies were obtained from Uwe Walldorf and were used at 1:1000, DAPI for nuclear staining was used at 1 μg/ml. Rat monoclonal antibody YL_1,2_ to detect freshly-made Tubulin [11] was used at 1:1000. For color conversion and interpretation of signal intensities, the OsiriX DICOM program was used [86].

The protocol for fluorescent in situ hybridization was adopted from [11], except that RNA probes were tagged with an Alexa Fluor 568 Signal-Amplification Kit (Invitrogen A11066).

### Embryo slicing, RNA extraction and PAT analysis

Embryos were mounted in OCT perpendicular to the blade of a Cryostat. Consecutive sections of 60 μm starting from the tip of 10 nc 13 wild-type embryos similar to the method as described by [54] were performed. RNA from the sections was isolated in TRIzol™ and quality-checked for degradation in a Bioanalyzer RNA pico chip. RNA was further concentrated using the direct-zol™ RNA MiniPrep Plus kit (Catalogue number #R2070) and a Zymo-spin™ IIICG column. DNase treatment was done directly on the column, followed by washing and elution with water. The length of the poly A tail was examined using the USB® poly A tail—Length Assay Kit (ThermoFisher, Catalogue number #76,455), following the manufacturer’s recommendation. The starting material had a RNA concentration in the range of 0.2 ng/μl. Two primer sets were used for amplification, one *bcd* gene-specific primer set giving a defined length and serving as control for integrity and concentration of the RNA (left side) and one primer set to amplify the gene of interest including the poly A tail (right side). The *bcd* gene-specific forward primer was 5’-CATTTTGCGCATTCTTTGACC-3’ and the *bcd* gene-specific reverse primer was 5’-TGTGTAGTTAGTCACAATTTACCC-3’. For poly A tail length determination, the *bcd* gene-specific forward primer again was 5’-CATTTTGCGCATTCTTTGACC-3’, identical to that described in [33], while the reverse primer was provided by the kit. PCR products were analyzed on a 2.5% agarose gel. The size of the amplified cDNA is 286 bp, 256 bp from *bcd* and 30 bp from the anchor.

### Data analysis

All images were recorded using a Zeiss LSM 710 confocal microscope. Images were post-processed with Adobe Photoshop and Adobe Illustrator. Image J was used to measure the length of the embryos, the distance between the anterior tip, the anterior border of each Eve stripe and the posterior border of Ems. All data was analyzed with Analysis of Variance (ANOVA) and two-tailed Student’s t-tests. Data are reported as means ± SE.

## Data Availability

The datasets generated during and/or analyzed during the current study are available from the corresponding author on reasonable request.

## Abbreviations

*bcd*: *bicoid*
*hb*: *hunchback*
*ems*: *empty spiracles*
*eve*: *even-skipped*
*run*: *runt*
*PAP2*: *poly* *A* *polymerase 2*
*wisp*: *wispy*
*CycB*: *Cyclin B*
MT: Microtubule
ARTS: Active mRNA transport, synthesis
SDD: Synthesis, diffusion, degradation
MTOC: Microtubule organizing center
LD: Lipid droplet
EL: Egg length
AA: Amino acid
